# Effects of TolC on the pathogenicity of porcine extraintestinal pathogenic *Escherichia coli*

**DOI:** 10.3389/fimmu.2022.929740

**Published:** 2022-08-18

**Authors:** Jin Hu, Dongfang Wang, Xingfa Huang, Yang Yang, Xin Lian, Wenjun Wang, Xiao Xu, Yulan Liu

**Affiliations:** ^1^ Hubei Key Laboratory of Animal Nutrition and Feed Science, Wuhan Polytechnic University, Wuhan, China; ^2^ Hubei Provincial Key Laboratory for Protection and Application of Special Plants in Wuling Area of China, South-Central University for Nationalities, Wuhan, China

**Keywords:** extraintestinal pathogenic *Escherichia coli*, tolerant colicin, pro-inflammatory cytokines, necroptosis, pathogenicity

## Abstract

Extraintestinal pathogenic *Escherichia coli* (ExPEC) is a well-known critical pathogenic zoonosis that causes extraintestinal infections in humans and animals by affecting their immune organs. Recently, research on the outer membrane protein of *E. coli*, tolerant colicin (TolC), a virulent protein in the formation of the ExPEC efflux pump, has been an attractive subject. However, the pathogenic mechanisms remain unclear. This study aimed to explore the role of TolC in the pathogenesis of the ExPEC strain PPECC42; a complementation strain (Cm-TolC) and an isogenic mutant (ΔTolC) were constructed. Loss of TolC drastically impaired the virulence of ExPEC in an experimental mouse model. ΔTolC showed a substantial decrease in the porcine aortic vascular endothelial cell (PAVEC) adherence, invasion, and pro-inflammatory response, in contrast to that of the wild type, with a reduced survival ratio in both the bacterial load and whole blood in mice. ΔTolC also showed decreased expression of necroptosis signals such as receptor-interacting protein kinase 1, phosphorylated mixed-lineage kinase domain-like protein, and mitochondrial proteins such as phosphoglycerate mutase family member 5. Our data suggest that TolC is closely associated with ExPEC pathogenesis. These results provide scientific grounds for exploring the potential of TolC as an effective drug target for controlling ExPEC infection, screening new inhibitors, and developing new drugs. This will allow for further prevention and control of ExPEC infection.

## Introduction

Extraintestinal pathogenic *Escherichia coli* (ExPEC) is an important pathogen responsible for a broad spectrum of infections and diseases in humans and animals (1–3). In general, ExPEC is not typically pathogenic when it colonizes the intestine. However, when ExPEC migrates to extraintestinal organs, it can induce life-threatening diseases such as septicemia, newborn meningitis, peritonitis, and urinary tract infections (4–8). ExPEC can effectively contaminate meat and enter the food chain (9). ExPEC infection is a major burden on the economy and the healthcare system, affecting both humans and animal husbandry. Therefore, establishing an effective and safe method for preventing ExPEC infections is of great importance.

ExPEC was recently discovered and has been frequently found in clinical samples in the pig industry (10). Isolates of ExPEC in large quantities originating from animals have been reported to have high antimicrobial resistance levels, and high virulence, thus indicating a high threat to public health (11–13). Johnson et al. (14) defined ExPEC strains as isolates of *E. coli* harboring a minimum of two or more virulent genes: *pap A/pap C* (P fimbriae), *sfa*/*foc* (S and FIC fimbriae), *afa*/*dra* (Dr-antigen-binding adhesins), *kpsMTII* (group 2 capsule synthesis), and *iutA* (aerobactin; iron acquisition system) (15, 16).

In addition to the TolC membrane protein, which is part of the efflux protein of the *E. coli* outer membrane (OEP), it plays a crucial role in the maintenance of both the structure and function of expulsion of many compounds (17, 18). TolC is essential not only for exporting large proteins but also for the efflux of small compounds. Several studies have previously shown that some TolC mutants are specifically resistant to colicin E1 (19) and are hypersensitive to detergents, antibiotics, and dyes (20, 21). Recently, there has been increasing interest in the correlation between TolC biofilm formation and efflux proteins (22, 23). Most studies on the efflux protein TolC have found that it plays an important role in biofilm formation in *E. coli*; whether TolC is associated with the regulation of ExPEC pathogenesis remains unknown. Therefore, this study aimed to characterize the role of TolC in the regulation of virulence of the ExPEC strain PPECC42 both *in vitro* and *in vivo*. This study will provide scientific evidence for exploring the potential of TolC as an effective antibacterial drug target for controlling ExPEC infections.

## Materials and methods

### Bacterial strains and culture

The wild-type (WT) ExPEC strain PPECC42 was isolated from diseased piglet lungs in 2006 in the Hunan Province of China (Accession No. NZ_CM003707.1 in GenBank). To construct a deleted TolC strain of ExPEC PPECC42, deletion of a 158-bp fragment was performed within the open reading frame (ORF) of TolC (24). Strains responsible for overexpressing TolC were constructed by electroporating the plasmid pHSG::tolC containing the full-length tolC gene of the ExPEC strain PPECC42 into an isogenic mutant (ΔTolC) (24). Clones on Luria–Bertani (LB) agar plates containing chloramphenicol within ΔTolC and a complementation strain (Cm-TolC) were selected and cultured on LB agar plates or in LB broth. When necessary, 25 μg/ml chloramphenicol was used. The cultures were incubated for 8 h at 37°C without shaking.

### Cell culture

Isolation, identification, and culture of porcine aortic vascular endothelial cells (PAVECs) were performed as previously described, with minor modifications (25, 26). PAVECs were obtained in small sheets after treatment of the aortic lumen (30 min, 37°C) with 0.1% type I collagenase (Sigma, St. Louis, MO, USA) in an M-199 medium (Gibco, New York, NY, USA) containing a penicillin–streptomycin solution (Gibco). Suspension and resuspension were performed. Suspension was performed by centrifugation at 100×*g* for 15 min, and during resuspension, PAVECs were resuspended in 5 ml M-199 medium containing 10% fetal bovine serum (Gibco, Victoria, Australia) and further cultured in a T-25 tissue culture plate (Costar, New York, NY, USA). Counting and viability detection of PAVECs were performed by trypan blue exclusion.

### *In vivo* infection studies

To further assess the role of TolC in virulence, we determined the survival rates of mice infected with WT, ΔTolC, or Cm-TolC. All animal experiments were performed in compliance with the Animal Welfare and Animal Experimental Ethical Inspection of the Wuhan Polytechnic University. Forty Kunming female mice (aged 5 weeks; n=10 per group) were randomly divided into four experimental groups. Mice in the infected groups were injected intraperitoneally with 200 μl of WT, ΔTolC, or Cm-TolC at 1×10^7^ CFU in phosphate-buffered saline (PBS). Ten mice in the control group were inoculated with PBS only, and their post-infection mortality was monitored for up to 10 days.

### Determination of viable bacteria in organs

Fifteen Kunming female mice (aged 5 weeks; n=5 per group) were initially infected by intraperitoneal injections of 200 μl of WT, ΔTolC, or Cm-TolC (1×10^7^ CFU in approximately 200 μl PBS). Mice injected with the same volume of sterile PBS were used as the controls. Furthermore, blood samples were acquired from the tail veins 12 h post-infection, and all mice were sacrificed simultaneously. We then evaluated the bacterial colonization in the blood, heart, lung, liver, and spleen samples. Samples were plated on tryptic soy broth (TSB) agar plates to determine the existence of viable WT, ΔTolC, and Cm-TolC strains within the homogenized organs (0.15 g per organ). Blood samples (100 μl) were plated. Finally, we counted the colonies and expressed them as CFU/g for the organ samples and CFU/ml for the blood samples.

### Histopathological analysis

The samples collected from the lungs and kidneys were fixed by immersion in 10% buffered formalin. After paraffin embedding, tissue sections (4-μm thickness) were stained with H&E and examined under a light microscope according to standard protocols.

### Bacterial assays

Bacterial assays were performed as previously described with minor modifications (27). Heparinized whole blood was collected from Kunming mice. The WT, ΔTolC, and Cm-TolC strains were harvested in the early stationary phase, washed twice with PBS, and diluted to 1×10^5^ CFU/ml. Subsequently, 50 μl of bacterial culture was mixed with 450 μl of fresh whole blood. The mixtures were then incubated for 1 h at 37°C with rotation. Aliquots of the samples were removed in 1-h intervals and plated to determine the number of viable bacteria. Using the formula [(CFU ml^−1^)_t=1h_]/[(CFU mL^-1^)_t=0h_] × 100, the results were expressed as the survival rate (%).

### Cell invasion and adherence assays

Cell invasion and adherence assays were performed as previously described, with minor modifications (28). Bacteria were cultured in brain heart infusion (BHI) for 6 h at 37°C, centrifuged, washed twice with PBS, and resuspended at 10^8^ CFU/ml in fresh Roswell Park Memorial Institute (RPMI) 1640 culture medium (Invitrogen, USA) without the use of antibiotics. Confluent monolayers of PAVECs grown in 24-well plates at 10^5^ cells/well were infected with 0.1-ml aliquots at a multiplicity of infection (MOI) of 100. To allow the cells to attach and invade further, the plates were centrifuged at 800×*g* for 10 min and incubated in RPMI 1640 medium for 2 h at 37°C with 5% CO_2._ The monolayers were washed three times with PBS, 100 μg/well of gentamicin and 5 μg/well of penicillin G were added, and the plates were then incubated for 45 min at 37°C with 5% CO_2_. To confirm that 100% of the exocellular bacteria were killed after antibiotic treatment, the culture medium from each well was removed, and the monolayers were washed thrice with PBS. The cells were then disrupted in each well by pipetting with 1 ml of deionized water. The number of viable bacterial cells was determined by plating appropriate dilutions of the lysates onto TSB agar plates.

Total cell-associated bacteria were also quantified for the cellular invasion assay, which was free of antibiotic treatment. Moreover, the invading bacteria were subtracted from the total cell-associated bacteria to determine bacterial adherence. All assays were performed in triplicate and repeated three times. Finally, the results were presented as the invasion or adherence rate relative to that of the WT, which was set at 100%.

### mRNA expression analysis by real-time PCR

Total RNA isolation, quantification, reverse transcription, and real-time PCR were performed as previously described (29). The primer pairs used for the amplification of target genes are presented in **Table 1**. The messenger RNA (mRNA) expression of target genes relative to the housekeeping gene (β-actin) was calculated using the 2^−△△CT^ method (30).

**Table 1:** Primer sequence used for real-time PCR.

Gene	Forward (5′– 3′)	Product length (bp)	Accession numbers
FADD	F: AAGTGTCTGACGCCAAG	101	XM_013987237.1
	R: CCTCCTGCTGTTCTTCC		
HMGB1	F: GCCTATCCATTGGTGATGTTG	260	NM_001004034.1
	R: TCCTCCTCCTCCTCCTCAT		
IL-1β	F: GCTAACTACGGTGACAACAATAATG	186	NM_214055.1
	R: CTTCTCCACTGCCACGATGA		
MLKL	F: TCTCGCTGCTGCTTCA	105	XM_013998184.1
	R: CTCGCTTGTCTTCCTCTG		
PGAM5	F: TCTTCATCTGCCACGCCAAT	104	XM_013992365.1
	R: GGTGATGCTGCCGTTGTTG		
RIP1	F: ACATCCTGTACGGCAACTCT	175	XM_005665538.2
	R: CGGGTCCAGGTGTTTATCC		
TNF-α	F: CTCTTCTCCTTCCTCCTGGTC	118	X57321.1
	R: ATGCGGCTGATGGTGTGA		
β-actin	F: TGCGGGACATCAAGGAGAAG R: AGTTGAAGGTGGTCTCGTGG	311	AJ312193.1

FADD, death domain; HMGB1, high-mobility group box-1 protein; IL, interleukin; MLKL, mixed-lineage kinase domain-like protein; PGAM5, phosphoglycerate mutase family member 5; RIP1, receptor-interacting protein kinase 1; TNF-α, tumor necrosis factor-α.

### Protein expression analysis by Western blot

PAVECs were seeded in six-well plates and incubated with WT, ΔTolC, or Cm-TolC for 4 h, followed by treatment with PBS. Cells were then lysed and subjected to Western blotting as previously described (31). Blots were incubated with primary antibodies against rabbit anti-receptor-interacting protein kinase 1 (RIP1) (#LS-B8214, LifeSpan), rabbit anti-total mixed-lineage kinase domain-like protein (t-MLKL) (#37705, Cell Signaling Technology), rabbit anti-phosphoglycerate mutase family member 5 (PGAM5) (#ab131552, Abcam), and mouse anti-β-actin (#A2228, Sigma Aldrich). The relative abundance of target proteins was expressed as the ratio of the target protein to β-actin.

### Statistical analysis

Data were analyzed using GraphPad Prism 5 (GraphPad, San Diego, CA, USA). Statistical analyses were performed using an unpaired Student’s *t*-test, and *p <*0.05 was considered statistically significant (*p < 0.05, **p <0.01, ***p <0.001, and ****p < 0.0001).

## Results

### Survival curves

**Figure 1:**
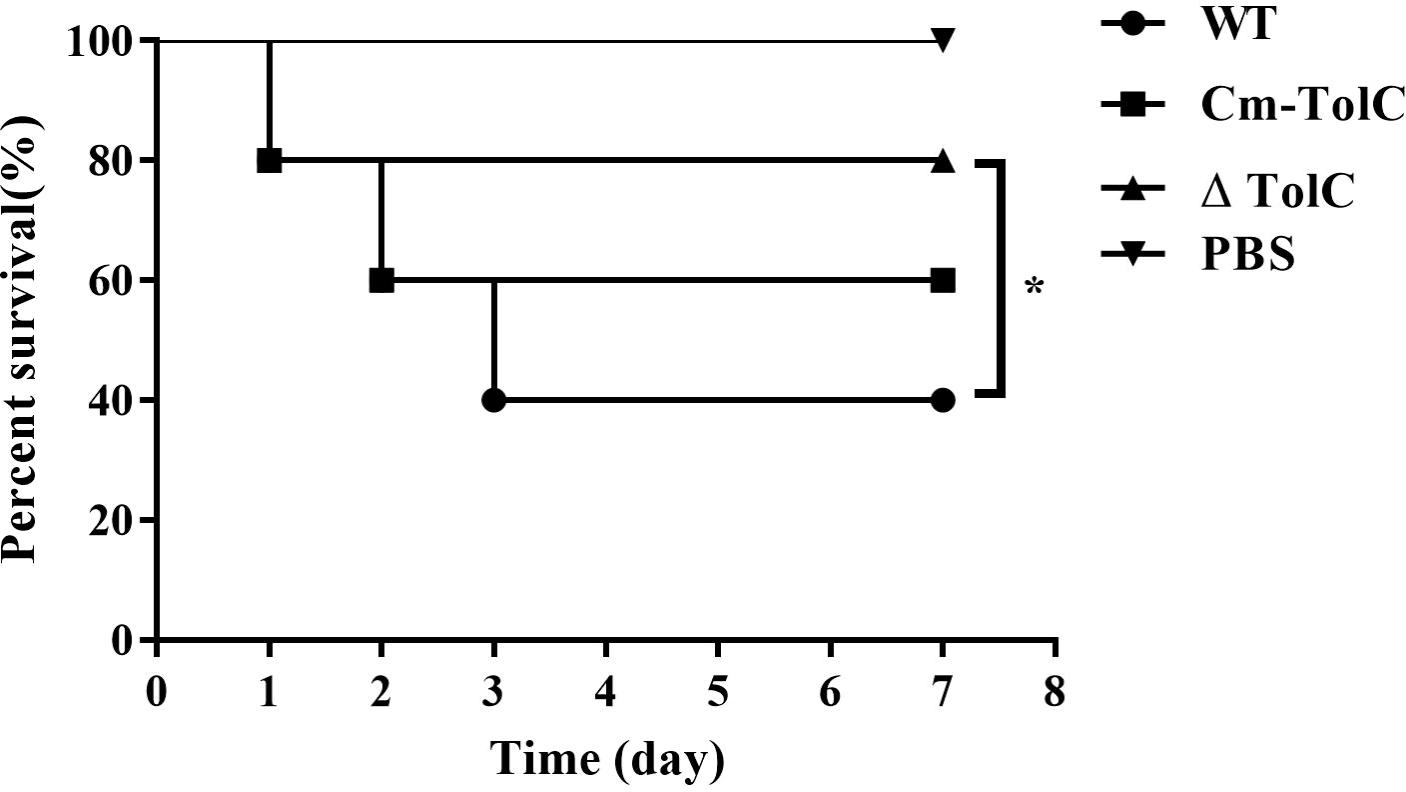
Survival curves for Kunming mice infected with the WT, ΔTolC, and Cm-TolC strains. Six-week-old Kunming mice were injected intraperitoneally with 1×10^7^ CFU of bacteria, and their survival was monitored over a 7-day period. Data were analyzed using the log rank test. Statistically significant difference is indicated as *p < 0.05.

Over the course of infection, mice infected with the WT at approximately 1×10^7^ CFU showed severe clinical signs such as rough coat, weight loss, eye abscess, and lethargy. Meanwhile, 10 mice infected with ΔTolC showed lethargy and slightly swollen eyes. In the WT group, only 40% of the mice survived until day 7 post-infection. However, mice in the ΔTolC group demonstrated an overall survival rate of 80% (**Figure 1**). These results indicated that deletion of the TolC gene significantly decreased the virulence of PPECC42 in mice (*p*<0.01).

### Histopathological of lungs and kidney

**Figure 2:**
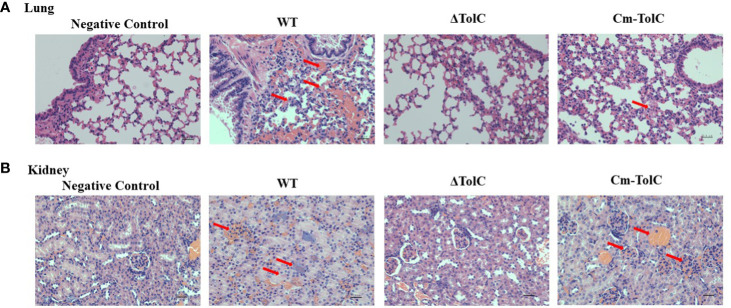
Histopathology of lungs and kidney of Kunming mice in different treatment groups. **(A)** Pathological examination of lungs tissues of the infected mice. **(B)** Pathological examination of kidney tissues of the infected mice. Arrowheads show the pathological changes. Representative images are shown for each group. Bars, 22.4 μm.

Histopathological analysis was performed to further explore pathological changes in the lungs and kidneys of the infected mice. The WT group showed hyperemia, hemorrhage, and alveolar space (**Figure 2A**). In contrast, minimal pathological changes were observed in the lung tissue of ΔTolC-infected mice. The kidneys of the mice in the WT group were showed inflammatory cells infiltration, glomerular enlargement, and vacuolization of renal epithelial cells (**Figure 2B**). However, these changes were not observed in the kidneys of ΔTolC-infected mice.

### Bacteria viable in organs

**Figure 3:**
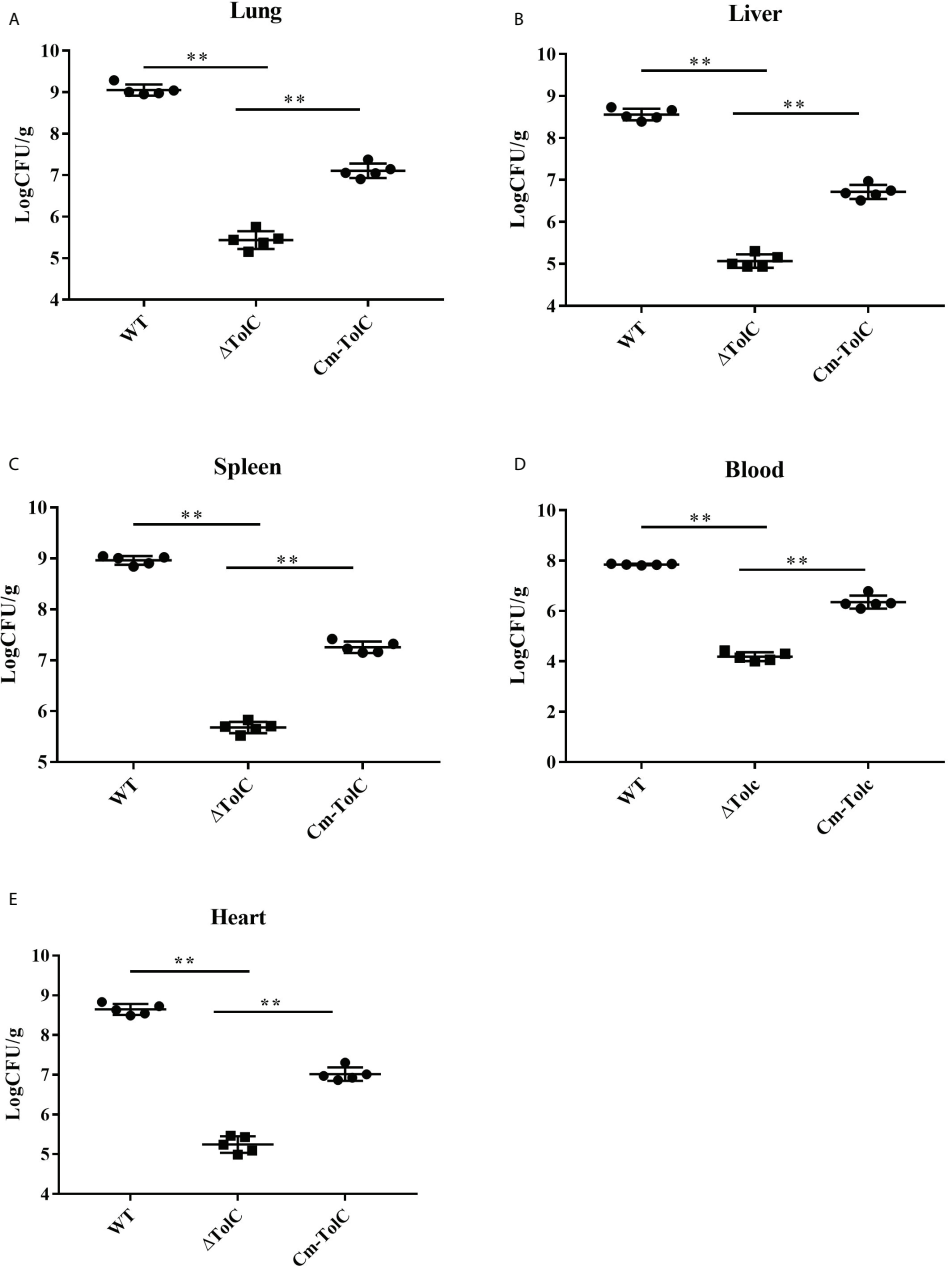
Distribution of bacteria in different organs from mice infected intraperitoneally with the WT, ΔTolC, and Cm-TolC strains. Bacterial loads in the lung **(A)**, live **(B)**, spleen **(C)**, and heart **(D)** are expressed as CFU per 0.15 g of tissue, and in the blood **(E)** as CFU per milliliter. Statistical analyses were performed by a repeated measures test with a two-tailed unpaired t-test. Statistically significant difference is indicated as **p ≤ 0.01.

To further explore the nature of reduced virulence, bacterial counts of the strains in the organs (heart, liver, spleen, and lung) of infected mice were determined at 12 h post-infection with sublethal doses. Bacterial counts from each tissue of ΔTolC-infected mice were significantly lower than those of WT-infected mice (**Figure 3**). This indicated that the TolC gene plays an important role in the pathogenesis of PPECC42.

### Mouse whole-blood bacterial killing assay

**Figure 4:**
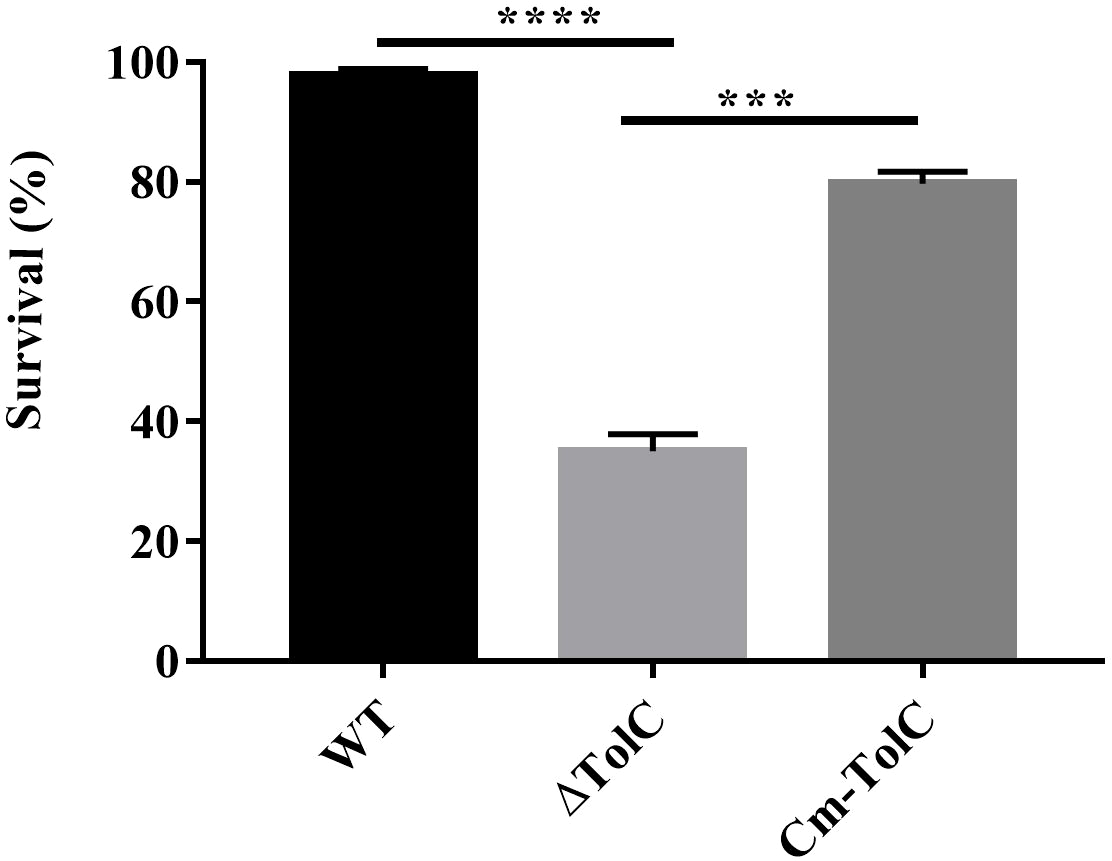
Effect of TolC on resistance to complement-mediated serum bactericidal activity. The resistance of WT, ΔTolC, and Cm-TolC to complement mediated serum bactericidal activity is shown. The results are expressed as the means of recovered bacteria per milliliter. Statistical analyses were performed using a two unpaired t-test. Statistically significant difference is indicated as ***p ≤ 0.001 and ****p < 0.0001.

To test the probability that PPECC42 TolC plays a role in the evasion of innate immune responses, we explored and examined the survival of the WT, ΔTolC, and Cm-TolC strains in whole blood collected from Kunming mice. After 1 h of incubation, the mean survival of ΔTolC were 33.01, 31.60, and 40.56. Those of the WT strain were 95.39, 98.61, and 99.07 (**Figure 4**). Moreover, survival of the Cm-TolC strain were restored relative to those of the mutant, but not to an extent where it reached the level of the WT strain (**Figure 4**). Moreover, the ΔTolC mutant in whole blood had a significantly slower survival rate than the WT (*p*<0.0001), further suggesting the effect of PPECC42 TolC on immune evasion.

### Adhesion and invasion in PAVECs

**Figure 5:**
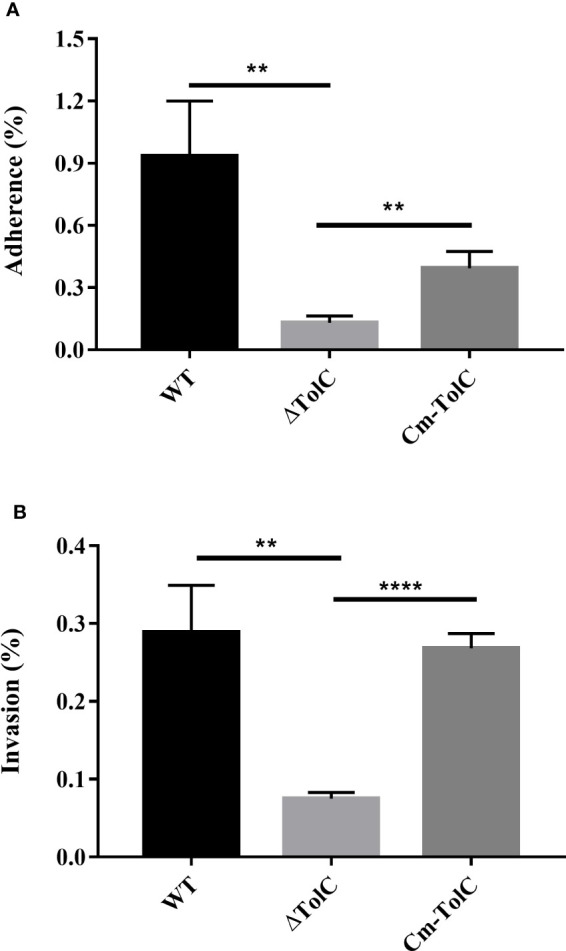
Adherence **(A)** to and invasion **(B)** into PAVECs by WT, ΔTolC, and Cm-TolC strains. The results are expressed as the means of recovered bacteria per milliliter. Statistical analyses were performed using a two unpaired t-test. Statistically significant difference is indicated as **p ≤ 0.01 and ****p ≤ 0.0001.

The adhesion and invasion of pathogenic bacteria to the mucosal surface is regarded as an important step in the process of infection. ΔTolC mutant levels of adherence and invasion into the PAVECs were significantly lower than those of the parent strain (*p*<0.01 for adhesion and *p*<0.01 for invasion) (**Figures 5A, B**), which was indicative of the probability of TolC in regulating some factors that contribute to cell adhesion and invasion.

### Pro-inflammatory and necroptosis responses in PAVECs

**Figure 6:**
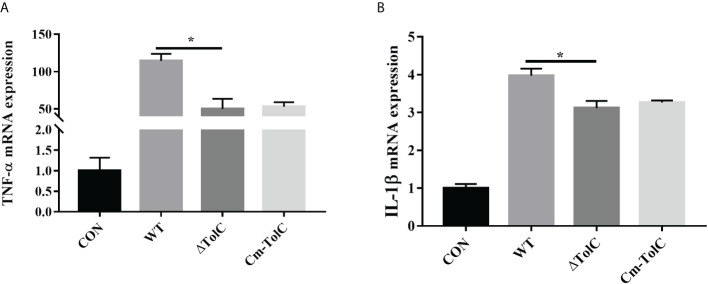
Induction of cytokine mRNA expression in PAVECs by stimulation with the WT, ΔTolC, and Cm-TolC strains. **(A)** mRNA expression of TNF-α. **(B)** mRNA expression of IL-1β. Cytokine mRNA levels were then determined by qRT-PCR. Statistical analyses were performed using a two unpaired t-test. Statistically significant difference is indicated as *p ≤ 0.05.

**Figure 7:**
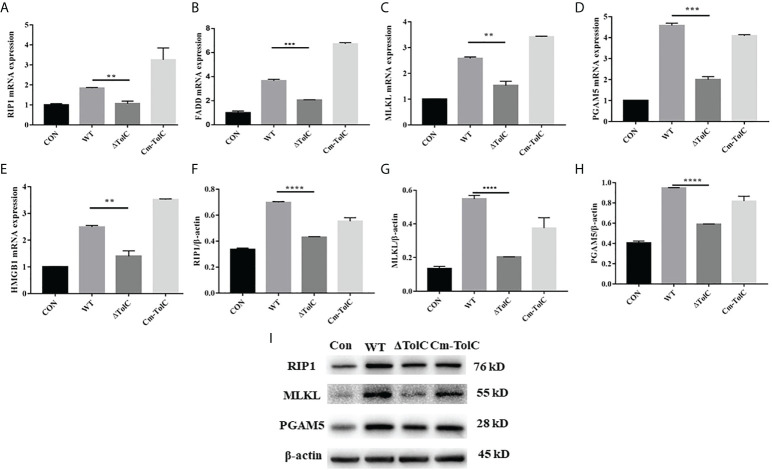
Effect of TolC on protsein abundance of necroptsis-related signaling components in PAVECs. **(A–E)** mRNA abundance of necroptosis signaling components in PAVECs. **(F–I)** Protein expression of necroptosis signaling components in PAVECs. Statistical analyses were performed using a two unpaired t-test. Statistically significant difference is indicated as **p ≤ 0.01, ***p ≤ 0.001 and ****p < 0.0001.

After the PAVECs were incubated with the WT, ΔTolC, and Cm-TolC strains, the levels of tumor necrosis factor-α (TNF-α) and interleukin-1β (IL-1β) were measured by quantitative real-time PCR (qRT-PCR). Deletion of TolC substantially decreased the pro-inflammatory ability of PPECC42 (**Figure 6**). These results indicated that TolC plays a crucial role in PPECC42-induced pro-inflammatory responses *in vitro*. Presently, in association with tissue injury and inflammation, necroptosis is regarded as a new form of cell death (32). To further identify the presence of necroptosis in intestinal PPECC42-infected PAVECs, we measured the mRNA and protein levels of important components of necroptosis, including RIP1, the death domain (FADD), MLKL, PGAM5, and HMGB1 (**Figures 7A–E**). Compared with PPECC42, ΔTolC reduced the mRNA levels of RIP1, FADD, MLKL, PGAM5, and HMGB1. Similarly, ΔTolC reduced the protein levels of RIP1, MLKL, and PGAM5 (**Figures 7F–I**). These results are shown in combination with the severity of the hepatic injury and inflammation.

## Discussion

In this study, we investigated the role of TolC in the pathogenesis of the ExPEC through mouse and cell experiments. Our results suggest that TolC is closely associated with ExPEC pathogenesis in mice. To induce disease, ExPEC must survive in the bloodstream after transmission *via* the respiratory tract. Thus, we compared the survival rate of the mutant and WT strains in whole mouse blood. The results of the whole-blood experiment showed that the ExPEC survival rate of the ΔTolC group was significantly reduced after 1 h. However, Mu et al. (33) reported that the ΔTolC had similar survival rate to the wild-type strain in specific pathogen-free (SPF) chicken serum. The reason for this discrepancy might be due to the different dependence of serum resistance on TolC expression between mice and chicks. The results of our *in vivo* colonization experiments also showed that ΔTolC displayed a significant reduction in bacterial colonization in tissues, including the heart, liver, spleen, and lungs. Similar to our data, Li et al. (34) reported that loss of TolC alleviated the clinical signs of pericarditis and spleen and liver enlargement, and decreased the pathogenicity to the host cells in chicks. Compared with the WT and Cm-TolC groups, the adhesion and invasion ability of the ΔTolC group to PAVECs was dramatically reduced. In agreement with our results, Buckley et al. (21) reported that disruption of TolC abolished the ability of *S. Typhimurium* to adhere, invade, and survive in both cell types. In addition, Mu et al. (33) reported that disruption of TolC significantly decreased the pathogenicity of Avian pathogenic *E. coli*. This suggests that the absence of TolC may lead to fewer bacteria colonization *in vivo* and cause less tissue damage to the host post-infection.

The elimination of pathogens is due to the activation of inflammatory responses, which generally benefit the host (35, 36). However, excessive inflammation is harmful and can lead to shock and organ failure (37). In our study, compared to the WT group, the mRNA expression of genes related to inflammation, such as *TNF-α* and *IL-1β*, was significantly downregulated in PAVECs infected with ΔTolC ExPEC. TolC has been shown to function as part of a T1SS for the delivery of *Francisella tularensis* effectors that alter host innate immune responses during infection (38). Taken together, a TolC-related function is required for ExPEC to activate proinflammatory responses.

Necroptosis has emerged as a vital pathway of programmed cell death in inflammation, immunity, and tissue homeostasis. Throughout necroptosis, interactions of RIP1 takes place *via* the RIP homotypic interaction motif (39, 40). Phosphorylation arises when RIP3 binds to the substrate MLKL (39). Furthermore, the RIP1/RIP3 necrosome has been suggested as an activator of PGAM5, leading to cell necroptosis (41). The release of intracellular damage-associated HMGB1 protein is due to cell rupture and necrosis, further promoting ongoing inflammation and secondary tissue injury (42). TolC is located in the bacterial outer membrane and can directly interact with host proteins to mediate the suppression of apoptosis upon infection by *F. tularensis* (38). Nowadays, the studies about the effect of TolC on necroptosis are lacking. Our results showed that mRNA expressions of *RIP1*, *MLKL*, and *PGAM5*, associated with *FADD* and *HMGB1*, were significantly downregulated. Furthermore, extremely downregulated expression of the necroptosis proteins RIP1, MLKL, and PGAM5 was observed in the ΔTolC group. Our data showed that a TolC*-*induced increase in pro-inflammatory cytokines causes tissue damage accompanied by necroptosis.

Our study had several limitations. First, to analyze the signaling pathways responsible for TolC-induced pro-inflammatory responses, inhibitors of RIP1, MLKL, and PGAM5 should be used. Second, the recombinant protein TolC should be analyzed by sodium dodecyl sulfate–polyacrylamide gel electrophoresis (SDS-PAGE) and Western blotting to determine whether TolC influences pro-inflammatory factors and necroptosis. Further investigation of the TolC-associated regulatory networks would be of great interest to further elucidate the mechanism of action.

In conclusion, our study demonstrates for the first time that TolC is important for the virulence of the ExPEC strain PPECC42. This study provides a scientific basis for exploring the potential of TolC as an effective drug target for controlling ExPEC infection, screening new inhibitors, and developing new drugs to better prevent and control ExPEC infection.

## Data availability statement

The original contributions presented in the study are included in the article/supplementary material. Further inquiries can be directed to the corresponding author.

## Ethics statement

The animal study was reviewed and approved by Wuhan Polytechnic University.

## Author contributions

Experimental design: YL and JH. Performance of experiments: JH, DW, XH, YY, XX, and XL. Data analysis: JH, YY, XL, and WW. Manuscript writing: YL and JH. All the authors approved the final version of the manuscript.

## Funding

This study was financially supported by the Science and Technology Research Program of Hubei Provincial Department of Education (No. B2021121).

## Conflict of interest

The authors declare that the research was conducted in the absence of any commercial or financial relationships that could be construed as a potential conflict of interest.

## Publisher’s note

All claims expressed in this article are solely those of the authors and do not necessarily represent those of their affiliated organizations, or those of the publisher, the editors and the reviewers. Any product that may be evaluated in this article, or claim that may be made by its manufacturer, is not guaranteed or endorsed by the publisher.
